# Clinical characteristics and antibiotic resistance profile of invasive MRSA infections in newborn inpatients: a retrospective multicenter study from China

**DOI:** 10.1186/s12887-023-04084-0

**Published:** 2023-05-25

**Authors:** Xia Wu, Chuanqing Wang, Leiyan He, Hongmei Xu, Chunmei Jing, Yinghu Chen, Jikui Deng, Aiwei Lin, Huiling Deng, Huijun Cai, Yiping Chen, Jinhong Yang, Ting Zhang, Qing Cao, Jianhua Hao, Yuanyuan Huang, Hui Yu

**Affiliations:** 1 Department of Infectious Diseases, Children’s Hospital of Fudan University, National Children’s Medical Center, Shanghai, 201102 China; 2 Department of Clinical Microbiology Laboratory, Children’s Hospital of Fudan University, National Children’s Medical Center, Shanghai, 201102 China; 3 Department of Infectious Diseases, Children’s Hospital of Chongqing Medical University, Chongqing, 400014 China; 4 Department of Clinical Laboratory, Children’s Hospital of Chongqing Medical University, Chongqing, 400014 China; 5 Department of Infectious Diseases, Children’s Hospital of Zhejiang University School of Medicine, Hangzhou, 310052 China; 6 Department of Infectious Diseases, Shenzhen Children’s Hospital, Shenzhen, 518038 China; 7 Department of Infectious Diseases, Qilu Children’s Hospital of Shandong University, Jinan, 250022 China; 8 Department of Infectious Diseases, Xi’an Children’s Hospital, Xi’an, 710003 China; 9 Department of Clinical Laboratory, Xi’an Children’s Hospital, Xi’an, 710003 China; 10 Department of Pediatric Infectious Diseases, Second Affiliated Hospital & Yuying Children’s Hospital of Wenzhou Medical University, Wenzhou, 325027 China; 11 Department of Clinical Laboratory, Second Affiliated Hospital & Yuying Children’s Hospital of Wenzhou Medical University, Wenzhou, 325027 China; 12 Department of Gastroenterology and Infectious Diseases, Children’s Hospital of Shanghai Jiaotong University School of Medicine, Shanghai, 200040 China; 13 Department of Infectious Diseases, Shanghai Children’s Medical Center of Shanghai Jiaotong University School of Medicine, Shanghai, 200127 China; 14 Department of Infectious Diseases, Kaifeng Children’s Hospital, Kaifeng, 475000 China; 15 Department of Pediatrics, Bethune First Hospital of Jilin University, Changchun, 130021 China

**Keywords:** Methicillin-resistant *Staphylococcus aureus*, Invasive infection, Clinical characteristics, Antimicrobial resistance, Neonates

## Abstract

**Background:**

Methicillin-resistant *Staphylococcus aureus* (MRSA) can cause invasive infections with significant mortality in neonates. This study aimed to analyze the clinical characteristics and antibiotic resistance profiles of invasive MRSA infections and determine risk factors associated with invasive MRSA infections in newborn inpatients.

**Methods:**

This multicenter retrospective study of inpatients from eleven hospitals in the Infectious Diseases Surveillance of Pediatrics (ISPED) group of China was performed over a two-year period (2018–2019). Statistical significance was calculated by applying the χ2 test or by Fisher’s exact test in the case of small sample sizes.

**Results:**

A total 220 patients were included. Among included cases, 67 (30.45%) were invasive MRSA infections, including two deaths (2.99%), while 153 (69.55%) were noninvasive infections. The invasive infections of MRSA occurred at a median age of 8 days on admission, which was significantly younger compared to 19 days in noninvasive cases. Sepsis (86.6%) was the most common invasive infection, followed by pneumonia (7.4%), bone and joint infections (3.0%), central nervous system infection (1.5%), and peritonitis (1.5%). Congenital heart disease, low birth weight infant (<2500 g), but not preterm neonates, and bronchopulmonary dysplasia, were more commonly found in invasive MRSA infections. All these isolates were susceptible to vancomycin and linezolid and were resistant to penicillin. Additionally, 69.37% were resistant to erythromycin, 57.66% to clindamycin, 7.04% to levofloxacin, 4.62% to sulfamethoxazole-trimethoprim, 4.29% to minocycline, 1.33% to gentamicin, and 3.13% were intermediate to rifampin.

**Conclusion:**

Low age at admission (≤8 days), congenital heart disease, and low birth weight were associated with invasive MRSA infections in neonates, and no isolates resistant to vancomycin and linezolid were found. Determining these risks in suspected neonates may help identify patients with imminent invasive infections who may require intensive monitoring and therapy.

**Supplementary Information:**

The online version contains supplementary material available at 10.1186/s12887-023-04084-0.

## Introduction

Methicillin-resistant *Staphylococcus aureus* (MRSA), which was first reported in the United States in 1961 and first described in the neonatal ward in 1981 [1], is a formidable pathogen that is commonly found in neonatal intensive care units (NICUs). The prevalence of neonatal MRSA colonization was reported in 3.9-8.4% of neonates, among whom one-forth developed MRSA infections [2–4], and 33-67% of *S.aureus* infections in neonates were caused by MRSA [5, 6]. MRSA outbreaks remain an ongoing concern in neonatal care and are increasingly complicated by high rates of resistance. MRSA can cause serious infections in the newborn, and has caused mortality in many countries throughout the world [7, 8]. While it is well known that neonates are vulnerable to invasive infection with MRSA, there is a real shortage of neonatal data on the profiles of MRSA infection and antimicrobial resistance rates.

Herein, we analyzed the clinical and antibiotic resistance profiles of invasive MRSA infections in neonatal from eleven hospitals within the Infectious Diseases Surveillance of Pediatrics (ISPED) group of China over a two-year period (2018–2019) and factors related to severity.

## Materials and methods

### Surveillance population

This study was conducted across eleven hospitals within the ISPED group of China between January 2018 and December 2019. There are a total of 314 newborns. According to the clinical manifestations and treatment response of the children, 94 cases that were considered to be colonized or contaminated by MRSA were excluded. A total of 220 MRSA infections were obtained. The total number of S. aureus infections was 659, and the proportions of MRSA infections were 33.38%. 220 cases of MRSA infection from the following hospitals: Children’s Hospital of Fudan University (67), Children’s Hospital of Zhejiang University School of Medicine (55), Children’s Hospital of Chongqing Medical University (18), Qilu Children’s Hospital of Shandong University (11), Shenzhen Children’s Hospital (10) Xi’an Children’s Hospital (10), Second Affiliated Hospital & Yuying Children’s Hospital of Wenzhou Medical University (10), Shanghai Children’s Medical Center of Shanghai Jiaotong University School of Medicine (10), Kaifeng Children’s Hospital (10), Bethune First Hospital of Jilin University (10) and Children’s Hospital of Shanghai Jiaotong University School of Medicine (9) were included in the analysis.

We reviewed the medical records of neonatal hospital inpatients with MRSA infections using a standardized data sheet, which included sex, age, underlying disease, infection site, clinical symptoms, treatments, prognosis, hospital stay length, and antibiotic resistance profile. All collected medical data were independently reviewed by two doctors. If multiple specimens from a single patient yielded MRSA-positive cultures, it was counted as a single case; however, strains from different specimen sources of the same patient were counted as different isolates. Species identification was performed by standard biochemical methods.

### Case definitions and ascertainment

Invasive MRSA infections were defined as clinical infections with isolation of MRSA from normally sterile body sites, such as blood, cerebrospinal fluid, pericardial fluid, pleural fluid, peritoneal fluid, bone, and joint fluid or other internal body sites.

Sepsis was defined as systemic inflammatory response syndrome (SIRS) associated with infection [9]. The SIRS in children was defined with the presence of at least two of the following four criteria, one of which needed to be abnormal temperature or leukocyte count: (1) cored temperature of >38.5℃ or <36℃; (2) tachycardia or bradycardia; (3) mean respiratory rate increased; (4) leukocyte count elevated or depressed for age or >10% immature neutrophils.

### Antimicrobial susceptibility testing

Antimicrobial susceptibility test (ASTs) was performed by dilution method (Vitek2 compact) and disk diffusion method. The ASTs breakpoint criteria of Clinical and Laboratory Standards Institute (CLSI) M100 S29 were adopted [10]. The ASTs were applied to penicillin, erythromycin, clindamycin, levofloxacin, sulfamethoxazole-trimethoprim (TMP-SMX), gentamicin, rifampin and minocycline. The dilution method (Vitek2 compact) was applied to penicillin, erythromycin, clindamycin, levofloxacin, sulfamethoxazole-trimethoprim (TMP-SMX), gentamicin, and rifampin, while the disk diffusion method was applied to minocycline.

### Reference strains

*Staphylococcus aureus* ATCC 25,922, ATCC 29,213, ATCC 29,212 were included to ensure reproducibility of the antibiotic susceptibility testing procedure.

### Statistical analysis

Statistical significance was calculated by applying the χ^2^ test or by Fisher’s exact test in the case of small sample sizes, using the SPSS statistics (Version 20) program. A *p*-value of < 0.05 was considered as statistical significance.

## Results

### Clinical features

Among 220 included cases, 138 (62.73%) of the neonates were male. The median patients’ age at admission was 16 days (range: 1 day to 30 days). Among these cases, 67 (30.45%) were invasive MRSA infections, including two deaths (2.99%), while 153 (69.55%) were noninvasive infections.

Invasive and noninvasive infections were compared (Table 1). No significant differences were found in sex. The invasive infections of MRSA occurred at a median age of 8 days from admission, which was significantly younger compared to 19 days in noninvasive cases (*p* = 0.000). Congenital heart disease and low birth weight infant (< 2500 g)(*p* = 0.008, *p* = 0.029, respectively), but not preterm neonates and bronchopulmonary dysplasia, were more commonly found in invasive MRSA infections. Compared with noninvasive MRSA infections, invasive cases stayed in hospitals longer (median: 32.5 days [interquartile range: 1.0–305.0 days] vs. median: 11.0 days [interquartile range: 3.0–153.0 days]; *p* = 0.000 by the Mann-Whitney U-Test) and had a greater mortality rate (2.99% vs. 0.00%).

**Table 1:** Characteristics of invasive versus noninvasive MRSA infections in neonates

	Invasive infections (n = 67)	Noninvasive infections (n = 153)	χ2	*p* value
Sex, M/F	42/25	96/57	0.000	0.993
Age at admission (days), median (IQR)	8.0(1.0–29.0)	19.0(1.0–30.0)	-5.889^*^	0.000
Underling disease				
Congenital heart disease	21(31.34%)	24(15.69%)	7.020	0.008
Preterm Neonates	9(13.43%)	12(7.84%)	1.686	0.194
low birth weight infant	5(7.46%)	2(1.31%)	3.907	0.029
bronchopulmonary dysplasia	7(10.45%)	14(9.15%)	0.091	0.763
Immunodeficiency	0(0.00%)	1(0.65%)	-	-
Hospital stay (days), median (IQR)	32.5(1.0-305.0)	11.0(3.0-150.0)	7.498^*^	0.000
Died	2(2.99%)	0(0.00%)	-	-

Data are presented as n (%), median (IQR).

*, Mann-Whitney U-Test, Z

Abbreviations: MRSA, methicillin-resistant * Staphylococcus aureus*; IQR, interquartile range

The clinical characteristics of patients with invasive MRSA infections are shown in Table 2. Sixty-seven patients (30.45%) had invasive MRSA infections, of which two infants died (2.99%). Septicemia (86.6%) was the most common invasive infection, followed by pneumonia (7.4%), bone and joint infections (3.0%), central nervous system (CNS) infection (1.5%), peritonitis (1.5%), sepsis with pneumonia and bone and joint infections (n = 1). The most commonly used antibiotics were vancomycin or combination with beta-lactam (76.1%), with linezolid being used in 10 cases. The cure rates were 90.2% and 92.9%, respectively.

**Table 2:** Clinical characteristics of patients with invasive MRSA infections

	Antibiotic use								
	Vancomycin			Linezolid			Fosfomycin		β-lactam
	Single drug	+β-lactam	+Isepamicin	Single drug	+fosfomycin	+β-lactam	Single drug	+β-lactam	Single drug
Sepsis (n = 58)									
Primary Sepsis (n = 18)	5	11	0	0	0	1	0	0	1
Sepsis with pneumonia (n = 25)	2	12	0	8	1	2	0	0	1
Sepsis with SSTI (n = 7)	3	4	0	0	0	0	0	0	0
Sepsis with bone and joint infections (n = 3)	0	3	0	0	0	0	0	0	0
Sepsis with CNS infections (n = 2)	0	2	0	0	0	0	0	0	0
Sepsis with pneumonia and CNS infections (n = 2)	0	1	0	0	0	0	0	1	0
Sepsis with pneumonia and bone and joint infections (n = 1)	1	0	0	1	0	0	0	0	0
Pneumonia (n = 5)	1	3	1	0	0	0	0	0	0
Bone and joint infections (n = 2)	1	0	0	0	0	1	0	0	0
CNS infection (n = 1)	1	0	0	0	0	0	0	0	0
Peritonitis (n = 1)	1	0	0	0	0	0	0	0	0
Treatment success rate (%)	86.7% (13/15)	91.7% (33/36)	0.0% (0/1)	100.0% (9/9)	0.0% (0/1)	100.0% (4/4)	0.0% (0/0)	0.0% (0/1)	0.0% (0/2)

Abbreviations: MRSA, methicillin-resistant *Staphylococcus aureus*; SSTI, skin and soft tissue infection; CNS, Central nervous system

We analyzed the clinical features of the two departed patients, a boy, and a girl. They were 1 day and 13 days old at admission, respectively. Both patients had underlying diseases of congenital heart disease. Their clinical symptoms included sepsis with pneumonia and sepsis with skin and soft tissue infection (SSTI), respectively. These two patients received vancomycin in combination with a beta-lactam for 20 days and 14 days, respectively. Unfortunately, the two patients experienced no improvement in the severity of the infection and eventually died.

### Bacterial identification and antibiotic susceptibility tests

**Fig. 1:**
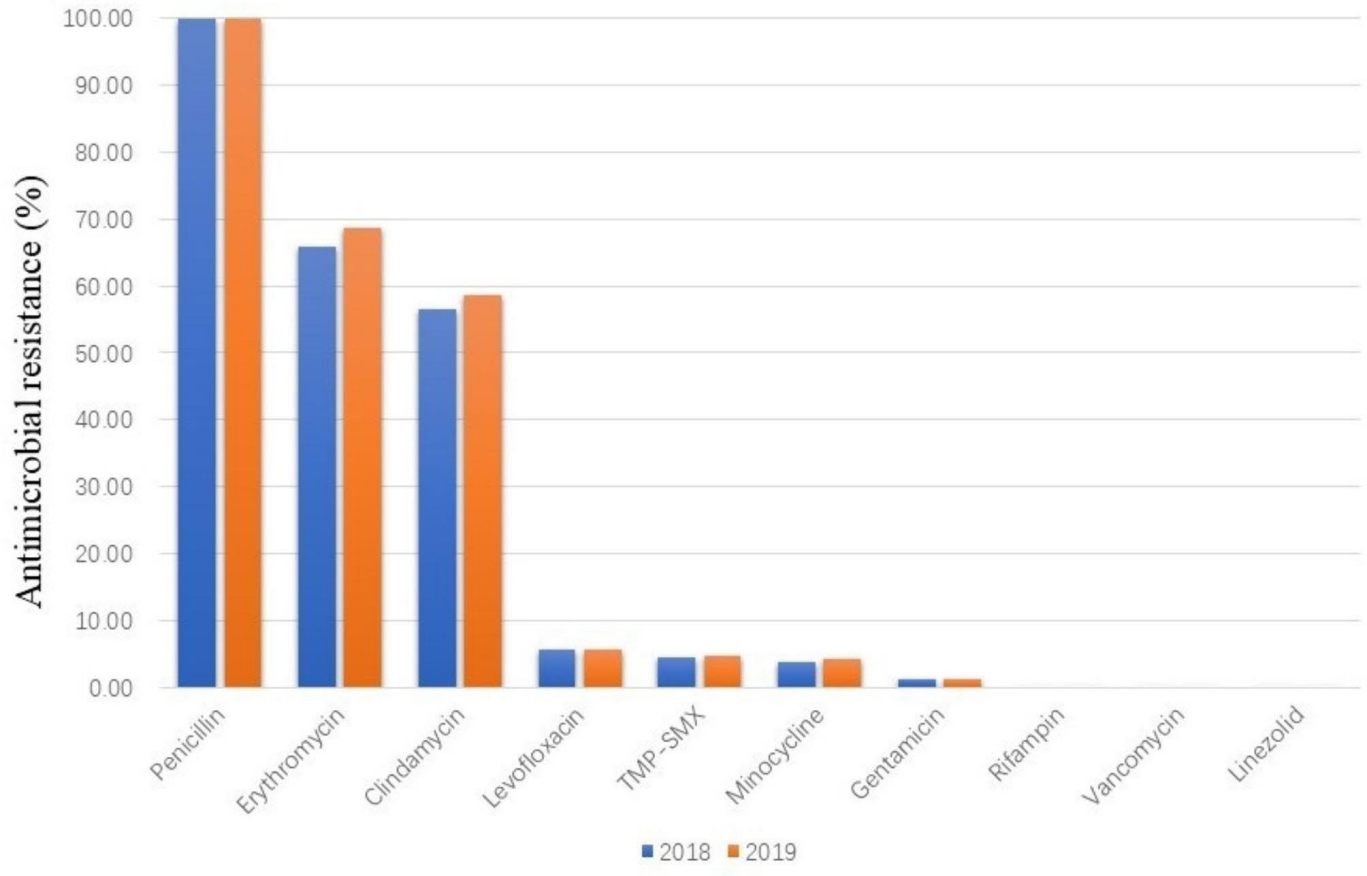
Profile of resistance to main antimicrobials (%) of MRSA isolates collected from 2018 to 2019

A total of 228 MRSA clinical isolates were obtained. All these isolates were susceptible to vancomycin and linezolid and were resistant to penicillin. Additionally, 69.37% of them were resistant to erythromycin, 57.66% to clindamycin, 7.04% to levofloxacin, 4.62% to sulfamethoxazole-trimethoprim (TMP-SMX), 4.29% to minocycline, 1.33% to gentamicin, and 3.13% were intermediate to rifampin (Fig. 1).

Of the MRSA isolated from invasive infections, 78.38% were resistant to erythromycin, 63.51% were resistant to clindamycin, 10.00% were resistant to levofloxacin, 5.56% were resistant to TMP-SMX, 7.69% to minocycline, and 1.35% were resistant to gentamicin. The erythromycin-resistance rate of the strains isolated from invasive infections was higher than that of the noninvasive infections strains (78.38% vs. 64.86%; *p* = 0.039; Table 3).

**Table 3:** Difference in Antibiotic resistance of MRSA isolated from invasive and noninvasive infections in neonates

Antibiotics	Invasive (n = 74)				Noninvasive (n = 154)				χ2	*p* value
	n	R (%)	I (%)	S (%)	n	R (%)	I (%)	S (%)		
Penicillin	74	100.00	0.00	0.00	154	100.00	0.00	0.00	-	-
Erythromycin	74	78.38	0.00	21.62	148	64.86	0.00	35.14	4.240	0.039
Clindamycin	74	63.51	2.70	33.79	148	54.73	0.68	44.59	1.559	0.212
Levofloxacin	70	10.00	1.43	88.57	143	5.59	0.00	94.41	1.393	0.238
TMP-SMX	54	5.56	0.00	94.44	119	4.20	0.00	95.80	0.000	0.706
Minocycline	13	7.69	0.00	92.31	57	3.51	0.00	96.49	0.000	0.465
Gentamicin	74	1.35	5.41	93.24	151	1.32	0.00	98.68	0.000	1.000
Rifampin	74	0.00	5.41	94.59	150	0.00	2.00	98.00	-	-
Vancomycin	74	0.00	0.00	100.00	154	0.00	0.00	100.00	-	-
Linezolid	74	0.00	0.00	100.00	154	0.00	0.00	100.00	-	-

Abbreviations: MRSA, methicillin-resistant *Staphylococcus aureus*; TMP-SMX, sulfamethoxazole-trimethoprim; R, resistant; S, susceptible; I, intermediate; R, resistant

The antibiotic resistance of MRSA strains isolated from different infection sites is summarized in Table 4. The levofloxacin-resistance rates of blood-derived MRSA strains were significantly higher than that of soft tissue-derived strains (15.38% vs. 3.06%, *p* = 0.009).

**Table 4:** Antibiotic resistance rates of MRSA from different site of infection

Antibiotics	Soft tissue		Blood stream		Respiratory		Others		χ^2^	*p* value
	n	R (%)	n	R (%)	n	R (%)	n	R (%)		
Penicillin	100	100.00	42	100.00	79	100.00	7	100.00	-	-
Erythromycin	99	63.64	42	73.81	74	74.32	7	71.42	2.790	0.425
Clindamycin	99	56.57	42	64.29	74	56.76	7	42.86	1.457	0.692
Levofloxacin	98	3.06	39	15.38	70	8.57	7	0.00	6.580	0.037^*^
TMP-SMX	74	2.70	32	9.38	62	4.84	5	0.00	2.950	0.229
Minocycline	34	5.88	7	14.29	27	0.00	2	0.00	0.000	0.439
Gentamicin	99	0.00	42	2.38	77	2.60	7	0.00	-	-
Rifampin	99	0.00	42	0.00	74	0.00	7	0.00	-	-
Vancomycin	100	0.00	42	0.00	79	0.00	7	0.00	-	-
Linezolid	100	0.00	42	0.00	79	0.00	7	0.00	-	-

*, the levofloxacin-resistance rates of blood-derived MRSA strains were significantly higher than that of soft tissue-derived strains (15.38% vs. 3.06%, χ^2^ = 6.903, *p* = 0.009). Abbreviations: MRSA, methicillin-resistant *Staphylococcus aureus*; TMP-SMX, sulfamethoxazole-trimethoprim; R, resistant; others, containing bone and joint, central nervous system, hydrothorax, ascites, pericardial fluid, urine

## Discussion

MRSA is a particularly threatening pathogen found in neonates. While invasive MRSA infections have been associated with high morbidity and mortality in NICU, the clinical features and risk of invasive MRSA infections in the neonate have not yet been fully described. Accordingly, we conducted this large retrospective study of neonatal MRSA infections with a relatively high rate of invasive diseases across various regions in China.

Sixty-seven neonates (30.45%) in our study developed invasive MRSA infections, and fifty-eight (26.36%) had sepsis. Over recent years, outbreaks of MRSA infection among neonates have been increasingly reported [11, 12]. Although skin and soft tissue infections (SSTIs) are common manifestations of MRSA infections in neonates, invasive diseases, such as bacteremia, necrotizing pneumonia, osteomyelitis, myositis, empyema, meningitis, and septic shock, were also identified and were often accompanied by complications [5, 13]. The first report on invasive MRSA infection, which was from the neonatal ward in the United States in 1981, described a patient who had persistent bacteremia and multiple bone osteomyelitis [1]. Subsequently, numerous invasive MRSA infections were reported in various regions [14]. In a ten-year retrospective study in the UK, including a period between 1993 and 2003, 27% of 30 neonates with *S.aureus* bacteremia had MRSA bacteremia [8]. In the United States, MRSA has become a significant cause of sepsis in neonates since 2003, accounting for 47% of neonatal bacteremia due to *S.aureus* [7]. The increasing incidence and more severe outcomes of MRSA bloodstream infections among neonates were observed by Dolapo O (from 24 to 55%) [15]. In 2014, Lim et al. reported a case of MRSA necrotizing pneumonia with empyema in healthy neonates, who finally accepted pneumonectomy [16]. Sepsis is an important cause of mortality in neonates. Previous studies have reported that the case fatality risk for neonatal MRSA sepsis ranges from 9.5 to 55% [6, 17].

Several risk factors associated with severe invasive MRSA infections have been studied. Preterm, premature rupture, low birth weight, and cesarean section delivery have been associated with increased risk of invasive MRSA infection among neonates in multiple studies [18–20]. Yet, the birth weight was in response to multiple factors, such as the maternal pre-pregnancy age, smoking, and multiple gestations. Therefore, further studies are needed to address this issue. Several studies reported that the attack rate of neonatal sepsis was significantly increased in infants in the presence of a maternal history of infectious diseases [9, 21]. In contrast to other studies, Denkel et al. reported no risk factors for transmission of MRSA from mother to infant [22]. This study found strong associations between low age at admission (≤ 8 days), congenital heart disease, low birth weight, malnutrition, and invasive MRSA infections. Poorly developed host defense mechanism, prolonged parenteral nutrition, and the use of gastrointestinal tract tube and central venous catheter placement increased the risk of invasive MRSA infection in neonates with low birth weight and malnutrition. Our results are in accordance with studies from the National Institute of Child Health and Human Development Neonatal Research Network [23].

Antibiotic susceptibility monitoring in NICUs is crucial for clinicians to opt for the most appropriate empirical antimicrobial therapies for neonates suspected of MRSA infection. Over the past 10 years, surveillance studies of MRSA infections in NICUs have reported high resistance rates to erythromycin, clindamycin, and ciprofloxacin, while the majority of MRSA isolates were susceptible to TMP-SMX, tetracycline, rifampin, linezolid, ceftaroline, chlorhexidine, and mupirocin [3, 24, 25]. So far, there are no reports of vancomycin-intermediate *S. aureus* (VISA) or vancomycin-resistant *S. aureus* (VRSA) infections in neonates, which were firstly reported in adults in 1996 and 2002 [26, 27]. In the present study, no strains showed to be resistant to vancomycin, linezolid, or rifampin. High resistance rates to erythromycin, clindamycin and occasional resistance rates to levofloxacin, TMP-SMX, and gentamicin were observed.

The use of antibiotic treatment is a particular concern because of the limitation in antibiotic classes among neonates. Intravenous vancomycin is recommended for children with invasive MRSA infections. Nevertheless, vancomycin monotherapy have signifcant limitations such as slow bactericidal activity, poor tissue penetration, uncertain optimal dosing, and vancomycin exposure in invasive MRSA is associated with reduced vancomycin susceptibility. Combination antimicrobial therapies has been advocated as an alternative strategy to improve patient outcomes. Combination therapy with vancomycin plus beta-lactams for MRSA bacteremia showed lower clinical failure rates, and the combination exposures significantly suppresses the development of VISA [28, 29]. Several studies have documented that adding a beta-lactam to vancomycin or daptomycin may help shorten bacteremia and avoid recurrences in patients with MRSA bacteremia [30]. Daptomycin is not routinely used in neonates because of a scarcity of literature on its efficacy and safety in infants. but numerous cases have shown the benefits and relative safety of daptomycin use in neonates [31, 32]. Daptomycin may be considered in cases of clinical failure with vancomycin. The combination of daptomycin plus beta-lactams has been shown to be more effective for the treatment of invasive MRSA infections, such as bacteremia and endocarditis, as they have a synergistic effect between them [33–35]. However, a randomized clinical trial from 2015 to 2018 showed that combining a beta-lactam with standard therapy with vancomycin or daptomycin was not associated with reduced treatment failure and mortality [36]. Meta-analysis suggested that the combination therapy could improve some microbial outcomes, but it could not reduce mortality [37, 38]. In our study, septicemia was the most common invasive infection, in which 53.7% were treated with vancomycin plus beta-lactams, and over 90.0% of them were cured. Future studies are warranted to determine the optimal combination regimen for the treatment of invasive MRSA infections. Additionally, a number of novel antimicrobials and adjunctive therapies are in development, including phage therapy, photodynamic treatment, and combinatorial therapeutic used with nanoparticles and Oil Compounds [39–43].

### Limitation of the study

Our study has several limitations. As this was a retrospective study, it was not possible to rule out confounding factors and to fully explore the clinical data. Information on the maternal history of bacterial colonization, infectious diseases, and obstetric risk factors are lacking. The major strengths of our data are multicenter nature of the study, and the fact that data of this type for neonatal invasive MRSA infections are relatively lacking. Future studies on invasive MRSA infections in newborns are needed to further verify our findings.

## Conclusions

In conclusion, the susceptibility of newborns may contribute to the outbreak of invasive MRSA infections. Invasive infections should be monitored in neonates with high-risk factors, such as low age at admission (≤8 days), congenital heart disease, and low birth weight. As MRSA has low resistance to vancomycin and linezolid, they are recommended in controlling invasive MRSA infections in neonates.

## Electronic supplementary material

Below is the link to the electronic supplementary material.

Supplementary Material 1

## Data Availability

All data generated or analysed during this study are included in this published article and its supplementary information files.

## Abbreviations

MRSA: Methicillin-resistant *Staphylococcus aureus*
ISPED: Infectious Diseases Surveillance of Pediatrics
CLSI: Clinical and Laboratory Standards Institute
TMP-SMX: Sulfamethoxazole-trimethoprim
NICU: Neonatal intensive care units
SSTIs: Skin and soft tissue infections
SIRS: Systemic inflammatory response syndrome
VISA: Vancomycin-intermediate *S. aureus*
VRSA: Vancomycin-resistant *S. aureus*.
